# Mucoadhesive polysaccharides modulate sodium retention, release and taste perception

**DOI:** 10.1016/j.foodchem.2017.07.134

**Published:** 2018-02-01

**Authors:** Sarah L. Cook, Samuel Woods, Lisa Methven, Jane K. Parker, Vitaliy V. Khutoryanskiy

**Affiliations:** a Department of Pharmacy, University of Reading, Whiteknights, Reading, Berks RG6 6AD, United Kingdom; b Department of Food and Nutrition Sciences, University of Reading, Whiteknights, Reading, Berks RG6 6AD, United Kingdom

**Keywords:** Mucoadhesion, Polymer, Salt, Tastant, Retention, Release, Perception

## Abstract

The mucoadhesion between polymeric substances and mucosal membranes, widely exploited in the pharmaceutics industry to prolong drug residence, has been investigated as a means of retaining taste or aroma molecules in the oral cavity. This study shows that the mucoadhesive properties of carboxymethyl cellulose, a commonly used polysaccharide in the food and pharmaceutics industry, can modify retention, release and perception of sodium over time. A three-part study was designed coupling *in vitro* retention using *ex vivo* porcine tongue, sensory perception with a trained panel and *in vivo* retention of sodium ions in human volunteers. The findings suggest that although salt perception is stunted in samples containing a random coil, ionic, mucoadhesive thickener, the retention of sodium ions in the mouth is prolonged due to the mucoadhesive nature of the polysaccharide. Not only has this study-investigated mucoadhesion of liquid formulations in the oral cavity but it is also the first to link the mucoadhesive nature of a commonly used polysaccharide to the organoleptic properties of a food.

## Highlights

• A potential role for mucoadhesive polysaccharides in food products is investigated.
• Mucoadhesion of a well-known food grade polysaccharide was established.
• Sensory tests show the mucoadhesive reduces saltiness in solution.
• Despite this reduction in perceived saltiness, the mucoadhesive retains more sodium ions for longer.

## 1 Introduction

Mucoadhesion describes the adhesive forces between a polymeric substance (a mucoadhesive) and a mucosal membrane in the body. The mucoadhesive strength between a polymer and mucosal surface will depend on many factors including the polymer characteristics and the target environment. In pharmaceutics, mucoadhesives can be incorporated into various formulations such as tablets, patches, films, sprays and viscous liquids containing an active pharmaceutical ingredient (API). The mucoadhesive polymer excipient can be designed to control the residence time and rate of release of the API. The mechanisms leading to mucoadhesion and the various techniques to assess the mucoadhesion of formulations have been described in the literature (Davidovich-Pinhas and Bianco-Peled, 2010, Nair et al., 2013, Peppas and Huang, 2004, Smart, 2014). However, mucoadhesion has not been fully exploited by the food industry as a means of retaining small molecules, such as tastants, at the mucosal surfaces in the mouth.

Mucoadhesion in the oral cavity has been investigated with a regard to enhancing delivery of a diverse range of APIs by the prolonged contact on these surfaces (Perioli et al., 2004, Perioli et al., 2004, Salamat-Miller et al., 2005, Yehia et al., 2009). Target areas for drug delivery in the mouth include buccal and gingival epithelia as these are typically thinner and non-keratinised. Various food grade polysaccharides are considered as mucoadhesives because they enhance retention and can control the release of APIs in the oral cavity. These include food grade polysaccharides such as carboxymethyl cellulose (Yehia et al., 2009), sodium alginate (Richardson, Dettmar, Hampson, & Melia, 2004) and pectin (Thirawong, Nunthanid, Puttipipatkhachorn, & Sriamornsak, 2007).

Polysaccharides are employed in the food industry for their use as thickeners, emulsifiers and stabilisers. They are commonly employed to mimic the functions that fat imparts to a food matrix in reduced fat, liquid or semi-solid products such as increased viscosity, lubricity and bulk. Gums such as xanthan, guar and carrageenan, starches, and modified cellulose derivatives such as carboxymethyl cellulose (CMC) and hydroxypropyl methylcellulose are frequently used for such products. Although polysaccharides increase viscosity of liquid and semi-solid foods their chemical and physical properties vary drastically. For example, CMC is a linear polysaccharide made of β 1 → 4 linked glucose units with some of the hydroxyl groups substituted with carboxymethyl groups to render it soluble in water. Starch, on the other hand, is a branched polysaccharide consisting of glucose units joined by α 1 → 4 glycosidic bonds in the form of amylose (helical) or amylopectin (linear). Unlike CMC, starch swells within granules, unless gelatinised, limiting the formation of interconnecting chains.

Many studies have investigated the impact on the sensory perception and *in vivo* aroma release when increasing liquid and semi-solid foods viscosity with polysaccharide thickeners (Boland, 2004, Cook et al., 2003, Han et al., 2014, Keršiene et al., 2008, Koliandris et al., 2008, Secouard et al., 2003). It is well known that an increase in viscosity results in a stunted perception of most tastants and some aromas. This is very apparent at the critical point where random coils of polymers in solution begin to overlap and pass one another, referred to as the coil overlap concentration (c^∗^) (Hollowood, Linforth, & Taylor, 2002). However, the temporal release and perception of these compounds, particularly the non-volatile components, is seldom investigated. Of these that have used temporal experiments, the adhesive nature of polysaccharides has never been investigated separately to perception and only seldom alluded to as a potential mechanism (Mälkki, Heiniö, & Autio, 1993).

Flavour balance is a challenge presented in low fat food formulations as the reduction of the hydrophobic matrix of a food results in the increased release of hydrophobic aroma compounds from food matrices. This results in an aroma release that peaks and rapidly falls compared to higher fat counterparts where the release is more uniform over time (Malone & Appelqvist, 2003). Furthermore, the relative increase in the hydrophilic component of the food can reduce the perception of hydrophilic tastants such as sodium (Boisard et al., 2014). Flavour perception is a combination of the senses of taste and smell, with tastants and aroma molecules having a complex relationship that results in signals transmitted to the brain interpreting the flavour of a food. It has been shown in numerous studies that perception of taste influences aroma perception, even when the in-nose aroma concentration stays the same (Cook et al., 2003, Koliandris et al., 2008). Therefore, if mucoadhesives can deliver tastants at a lower rate over time, then aroma perception may be adjusted accordingly, resulting in a product with a flavour profile like that of a high fat product.

Lian, Malone, Homan, and Norton (2004) and Malone and Appelqvist (2003) attempted to prolong aroma delivery using gelled emulsion particles of calcium alginate. The results suggest that aroma release can be controlled by particle size. Emulsions and encapsulation of aromas have been widely researched, however, utilising mucoadhesion to prolong flavour delivery is a relatively novel concept. For the past few decades mucoadhesion has been researched in relation to pharmaceutical applications, however, more recently the potential for their use in food products to prolong flavour delivery has been considered (Le Révérend et al., 2010, Malone et al., 2003, Modh and Bakalis, 2011). This current study investigates the temporal retention, release and subsequent perception of a tastant, sodium chloride, in a model liquid food prepared containing two different polysaccharide thickeners and water. Firstly, the retention of matrices was tested on *ex vivo* porcine tongue to determine differences in residence time between each matrix. Mucoadhesion on the dorsal mucosa of the tongue has been reported in only one study to date which investigated the binding of different milk proteins to distinct areas of the tongue in an attempt to explain negative sensory attributes such as drying (Withers, Cook, Methven, Godney, & Khutoryanskiy, 2013). Therefore, this current study is the first to develop a method for assessing the adhesion of viscous polysaccharide solutions to *ex vivo* porcine tongue tissue.

We are the first to show that food grade mucoadhesives are retained on the tongue *in vitro*, alter the temporal perception of saltiness over time compared to non-mucoadhesives, and prolong sodium retention in the mouth despite a reduction in perception. Perception data was collected after consuming samples by a progressive profiling method to understand changes in perception over time. Furthermore, an *in vivo* retention experiment was developed to ascertain the differences in sodium levels retained by the mucoadhesive sample compared to non-mucoadhesive samples. Our hypothesis is that mucoadhesives may retain tastant and aroma molecules, extending the residence time in the oral cavity, delaying release and prolonging flavour perception.

## 2 Methods

### 2.1 Materials

The 3 matrices were prepared for all parts of this experiment; they were all aqueous solutions made with deionised water, or deionised water plus sodium carboxymethyl cellulose (CMC) as the mucoadhesive polysaccharide, or an amylase resistant starch (Nutrilis brand, Boots UK Ltd). The CMC used was kindly provided by Akucell upon request (sample code: AF0305, molecular weight of 140 kDa and a substitution degree of 0.8). The starch was purchased from a local Boots store to be used for thickening liquids for patients with dysphagia. It is a modified maize starch resistant to amylase due to its composition with more amylose units than amylopectin. Other minor ingredients in the amylase resistant starch are maltodextrin, xanthan gum, tara gum and guar gum.

The aqueous samples were freshly prepared on the day that they were used for analysis. Both CMC and starch were dispersed in deionised water to obtain a final concentration of 2.6% (w/w). CMC samples were prepared on the morning before experiments and left in the fridge for at least 3 h to remove air bubbles. Starch and water samples were prepared no longer than 30 min before commencing experiments to prevent the starch from thinning. All samples contained the same concentration of sodium (final concentration 0.18% Na^+^ or 786 μM) either from NaCl salt added or Na^+^ inherently present in the polysaccharide. The CMC contains a high amount of Na^+^ to make it soluble in water. Flame photometry (Economical Flame Photometer; 230 VAC, 50/60 Hz) was used to determine the amount of Na^+^ in CMC (51.5 mg/g) and therefore, the amount of NaCl added to these samples was adjusted to account for this inherent sodium concentration. This ensured that the dosage of sodium in each matrix was the same, but the amount of accompanying chloride was different.

The viscosities of the CMC and the starch sample were determined using a TA AR2000 rheometer with 40 mm parallel plate geometry (TA Instruments, Herts, UK). After the initial amplitude sweep to determine the linear viscoelastic regions of the samples, the amplitude was set to 1% strain and frequency sweeps were then carried out to determine the complex viscosity over increasing frequency (Fig. S1a & b). Various concentrations of CMC were measured to match the 2.6% (w/w) starch viscosity (55 mPa.s) at a shear rate of 50 rad/s (Fig. S3) as this is typically quoted as the shear rate in the mouth (Richardson et al., 1989, Wood, 1968).

### 2.2 Ex vivo retention experiments

A dynamic retention method previously developed by Khutoryanskiy and coworkers (Cave et al., 2012, Cook et al., 2015, Irmukhametova et al., 2011, Withers et al., 2013) was adapted for this experiment. The retention experiment allows indirect quantification of the amount of sample retained on a mucosal surface after being repeatedly washed with an artificial eluent. To visualise retention of the sample sodium fluorescein (0.01%) was added to the solutions prior to placement on the tissue. For this experiment, *ex vivo* porcine tongue was used as the mucosal surface and an artificial saliva (AS) formulation was used as adapted from Madsen, Sander, Baldursdottir, Pedersen, and Jacobsen (2013), as the eluent. This AS recipe was found to best simulate the retention profile achieved with real human saliva (Madsen et al., 2013). The AS was comprised of CaCl_2_ (4 mM), KCl (10 mM), NaHCO_3_ (2 mM), NaCl (7 mM), KH_2_PO_4_ (6.7 mM) and pig gastric mucin (2.5 % w/v) (Sigma Aldrich Poole, UK).

#### 2.2.1 Tissue preparation

Pig tongues were collected up to 24 h post slaughter from P & D Jennings (Hurst, UK) butchers where they were kept at −4 °C, and kept on ice during transit (20 min). Most connective tissue and muscle was removed from the underside of the tongue and the epithelial layer was kept in airtight bags at −20° (until required). The dorsal of the tongue is covered with a specialised epithelium consisting of keratinised and non-keratinised regions and many protrusions and crevices due to the ubiquitous papillae. The structure of the mucosa varies significantly in the different areas of the tongue so the front, rear and side portions were selected based on their differing morphologies. When required, the tissue sections were thawed at room temperature and cut into 1 cm^2^ sections (around 2 mm thick). These sections were glued mucosal side up onto microscope slides in order to enable handling of the tissue.

#### 2.2.2 Retention procedure

The polysaccharide samples were mixed with sodium fluorescein stock (1% sodium fluorescein in deionised water) for a final concentration of 0.01% (w/v). This addition of fluorophore allowed the visualisation and quantification of fluorescence under a fluorescent stereomicroscope (Leica MZ10F). After conditioning the tissue with 1 mL of AS, 30 μL of sample was applied to the mucosal surface with a syringe and allowed to equilibrate for 30 s. A picture of the unperturbed sample on the tissue was taken under the fluorescent microscope at this point, which would later be referred to as wash 0 or 100% fluorescence. The tissue was then placed on a plastic slide angled at 45 ° and washed with 20 mL AS, controlled by a syringe in an automatic pump set to 6 mL/min. At 1, 2, 3, 5, 10, 15 and 20 mL the flow of eluent was stopped and images were taken under the fluorescent microscope. The rate of salivary production in the mouth is estimated to be around 1 mL/min dependent on the stimulation (Fenoli-Palomares et al., 2004, Gaviao et al., 2004), therefore, this could be thought of as up to 20 min residence time in the mouth. The tissue was kept in an incubator set to 37 °C whilst being washed with eluent. Although this method simulates the oral cavity conditions to a certain extent, tongue and mouth movements cannot be simulated and therefore the residence time is unlikely to be as long *in vivo*. The fluorescent pictures were analysed using ImageJ software (National Institutes of Health) to quantify the intensity of fluorescence after each wash. Each sample and each area of the tongue was repeated three times on three different pig tongues. The WO_50_ values were calculated from the retention results. These WO_50_ values represent the volume (mL) of artificial saliva required to wash off 50% of the fluorescent sample (Mun, Williams, & Khutoryanskiy, 2016).

### 2.3 Sensory perception

The University of Reading screened and trained sensory panel of 11 people were trained to assess three attributes in the samples using a progressive profile method. After initial exposure to the samples, the panel decided on the attributes saltiness, adhesion and mouthcoating to best describe the samples. Panellists were trained on the saltiness attribute with a range standard samples that varied in concentration. They were given 0.4% NaCl in water as their extreme anchor. Two more standards 0.2% and 0.1% were given that were approximately 50% and 25% of the line scale. These were given to the panellists on several occasions to familiarise themselves with the scoring intensities. Adhesion was defined as the stickiness of the sample to the roof of the mouth and mouthcoating was defined as the feeling of something present on the mouth lining.

Progressive profiling produces a time-dependent descriptive profile showing the intensity of attributes over specific time period during or after consumption. The test was made in Compusense using standard unstructured line scales (scaled 0–100) (Fig. S4). In this experiment, the progressive profiling took place after the sample was swallowed in order to gather insights into the influence of adhesion on salt perception. Panellists were given 5 mL of each sample in opaque shot glasses and asked to score the attributes immediately after swallowing. They were then instructed to sit quietly and swallow a consistent number of times (dependent on the panellists individual defined times in 1 min), predetermined during training, for 20 s until the next scoring session in the progressive profile. Panellists took an average of 10 s to score the samples at each time point and therefore the time interval between scores was, on average, 30 s. Compusense collected data and the raw data was exported and analysed in SPSS. Panellists rinsed their mouth thoroughly with water for 2 min between samples.

### 2.4 *In vivo* sodium retention

An *in vivo* retention study was designed to determine the actual amounts of sodium retained in the mouth over time. It is well known that mucoadhesives retain small compounds at mucosal sites, hence, it was hypothesised that this would be the case with sodium chloride. Five participants were recruited, 1 female and 4 males, between the ages of 22 and 30. Ethical approval was sought and granted by the University of Reading’s School of Chemistry, Food and Pharmacy ethics committee prior to experiments (project code 27/15). Participants were asked to brush their teeth and rinse their mouth thoroughly with filtered water 15 min before they started each session. Each sample was tested in triplicate so each data point reported was a mean of 15 individual saliva collections.

#### 2.4.1 Saliva collection

For each session, the participants were given one of the three matrices containing salt each session of the experiment. Compusense software was used for timing each experiment and the breaks between each sample. For each sample, the participant was presented with 5 mL and asked to hold the sample in the mouth for 10 s before spitting out the sample into a disposable spittoon. To avoid excessive consumption of sodium chloride participants spat out the sample instead of swallowing. This first expectoration was not measured as this was in place of the participants swallowing. After this initial spitting, a timer started and once this had finished, the participant was prompted to scrape their tongue with their teeth and rid their whole mouth of saliva into a pre-weighed, appropriately labelled tube that would later be analysed. The timer counted down from either 5, 30, 60, 120, 180, 240 or 300 s to gather measurements of sodium retained at each of these time points. For every time point, a new sample was presented to the participant in order to accurately measure how much would be retained at each time point over the total 5 min period. There was at least a two min break between each sample in the series. Timings were randomised and swallowing was controlled during each experiment so that each individual participant was swallowing the same amount of times for each sample and all time points. Due to individual variances of saliva production the number of swallows per person was different.

#### 2.4.2 Analysis of sodium in saliva

The tubes were weighed before and after collection in order to determine the amount of saliva collected. The saliva samples were diluted with 40 mL deionised water and agitated so the sodium content could be measured by flame photometry set for sodium detection. Sodium chloride standards were used for a calibration curve ranging from 0 mg/L to 10 mg/L Na^+^ (Fig. S1) as this was in the linear range. A blank saliva sample was taken each day before experiments started to measure the sodium present in resting saliva. These blanks were averaged over the 9 sessions to give a value for baseline sodium content of each participant’s saliva. This was then subtracted from the results obtained from the experiments.

### 2.5 Statistical testing

For all experiments two way repeated measures ANOVA was used in the statistical analysis software, SPSS (IBM software). Bonferroni adjustments were made for multiple comparisons of time points. Fisher’s Least Significant Difference was used when comparing between the three matrices.

## 3 Results & discussion

### 3.1 *In vitro* retention of solutions

**Fig. 1:**
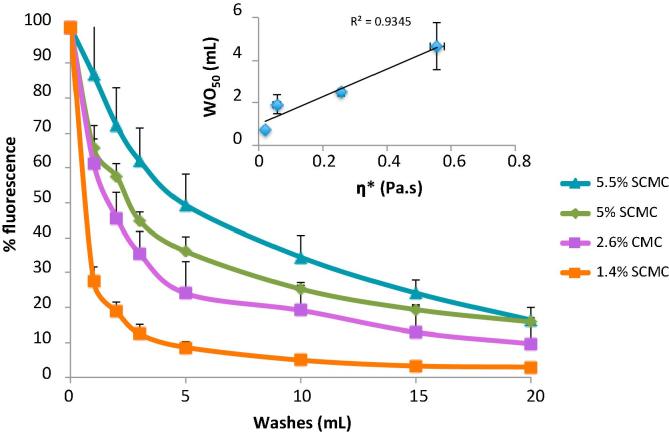
Wash off profile of various concentrations of CMC on the front of *ex vivo* porcine tongue. Each point represents the mean of 3 repeats of different tongues and is the percentage of fluorescence retained after washing with artificial saliva. Inset graph is the complex viscosity (η^*^) in Pa.s plotted against the amount of artificial saliva (mL) it took to wash off 50% of the sample (WO_50_).

Fig. 1 shows the retention profile of CMC at several concentrations. As the concentration of CMC increased in the sample, the viscosity also increased (Fig. S2). This is reflected in the retention profiles obtained (Fig. 1) where the least viscous sample (1.4% (w/w) CMC) was the least retentive followed by 2.6% (w/w), 5% (w/w) and 5.5% (w/w). Fig. 1 (inset) shows the linear relationship between complex viscosity (η^∗^) and WO_50_ values. Therefore, these results suggest that the retentive ability of the sample is viscosity dependent. Rheology results showed that CMC is relatively non-shear thinning at the concentrations below 5.5% (Fig. S2) and may explain the extended residence time on the mucosa. Although viscosity can be quoted at a single shear rate, the shear behaviour of the sample will be an important factor when considering the impact on mucoadhesion and retention of molecules.

**Fig. 2:**
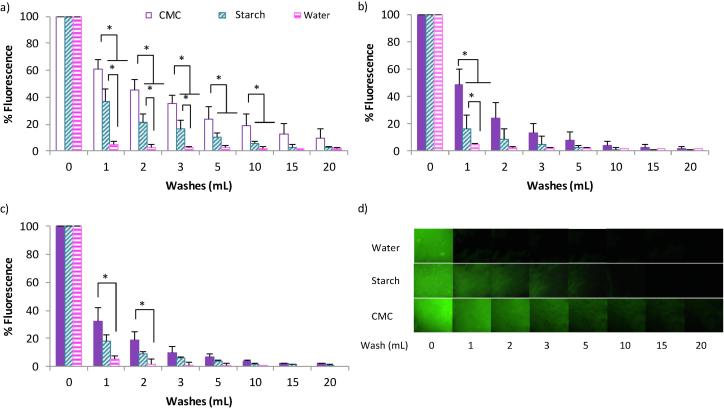
*In vitro* retention profiles of samples on the front (a), rear (b) and side (c) of *ex vivo* pig tongue and example fluorescent images (d). Significance value of p < 0.05 is represented by * between respective groupings (n = 3). Error bars are ±standard deviation. Fluorescence intensity of the retained polysaccharide was quantified by ImageJ software after being washed with artificial saliva up to 20 mL.

**Fig. 3:**
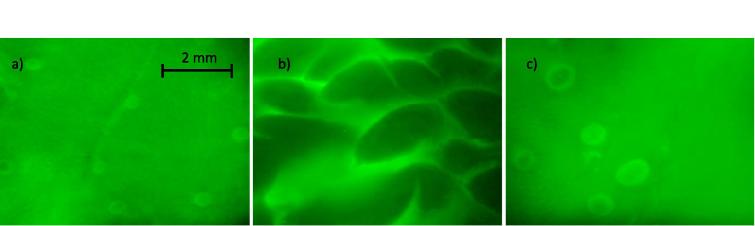
Fluorescence images of the differing morphologies of the different areas of the tongue. The font (a), rear (b) and side (c) of *ex vivo* porcine tongue after 0.1% sodium fluorescein was placed onto the different areas of tissue.

Fig. 2 shows the retention profiles of the CMC, starch (matched viscosities) and water samples on different areas of *ex vivo* pig tongue (Exemplary images Fig. 2d). The different areas of the tongue have different retention profiles with the front of the tongue retaining the polysaccharide matrices longer than the rear and side of the pig tongue. This is in accordance with previous results investigating milk protein retention on different tongue areas (Withers et al., 2013). This is probably due to the morphology of the front surface of the tongue, as it possesses a high density of fungiform and filiform papillae that increase the surface area and surface roughness, facilitating mucoadhesion. The rear of the tongue has larger protrusions and the side is mostly smooth, non-keratinised tissue with few papillae present. Fig. 3 shows some exemplar fluorescent photographs of the three areas highlighting the differing morphological surfaces.

As a control, sodium fluorescein in water was applied to the tissue and washed off. Fig. 2 shows that this solution was not retained on any of the areas of the tongue after the first wash with 1 mL AS. This shows that the dye is not being retained on the tissue without the presence of the polysaccharide. The starch sample was retained on the tongue longer than the water sample and this is most likely due to viscosity factors. On the front of the tongue most the sample was washed off after 5 mLs, whereas for the rear and side of the tongue 3 and 2 mL was sufficient, respectively. CMC on the other hand was still visible after 20 mL of AS washing on the front of the tongue.

During these experiments the shear force that the sample is put under is that from the droplet encountering the tissue. The shear rate that the sample viscosities were matched at was very high in order to emulate the reported conditions in the mouth. Therefore, at lower shear rates there is a large discrepancy between viscosities, with starch having a much higher viscosity than CMC (Fig. S2). Despite this, it was found that CMC was retained for longer than starch on the front of the tongue with a similar trend in the other areas. This suggests that viscosity is not the only driving factor for mucoadhesion, though this study (Fig. 1) and previous studies have shown that an increase in viscosity does result in enhanced mucoadhesion. The solubility of a polymeric substance in the mucosal secretion will also play an important role in the mucoadhesion observed. In this study both polysaccharides are hydrophilic and will, therefore, be soluble in saliva, which has a neutral pH.

There are many possible reasons why CMC is more retentive on the tongue mucosa than starch. Starch is a shear thinning polysaccharide used for its thickening properties in a range of liquid and semi solid food applications. Starch was chosen as a negative control for mucoadhesion in this experiment as it thickens solutions whilst being relatively non-adherent to the mucosal surface of the mouth, as illustrated by the *in vitro* retention (Fig. 2). Starch has a granular structure in solution where its polymer chains swell and form colloidal hydrated particles that exhibit limited chain entanglement (Mackley et al., 2013). Nutrilis is a modified form of starch, however, it still exhibits a granular, swollen texture rather than a continuous network of polymer chains (Mackley et al., 2013). This granular structure will affect the ability of the polymer chains to interpenetrate within the mucus layer to form physical entanglements with mucin, promoting adhesion. Conversely, the CMC polymer chains can settle into the micro cracks (papillae) that are present on the surface of the tongue leading to an increased polymer – surface interface. Furthermore, CMC is an anionic polysaccharide due to the presence of COO^−^ groups. This will contribute to mucoadhesion through hydrogen bonds and van der Waals forces with the mucin oligosaccharide side chains.

### 3.2 Sensory perception: saltiness

**Fig. 4:**
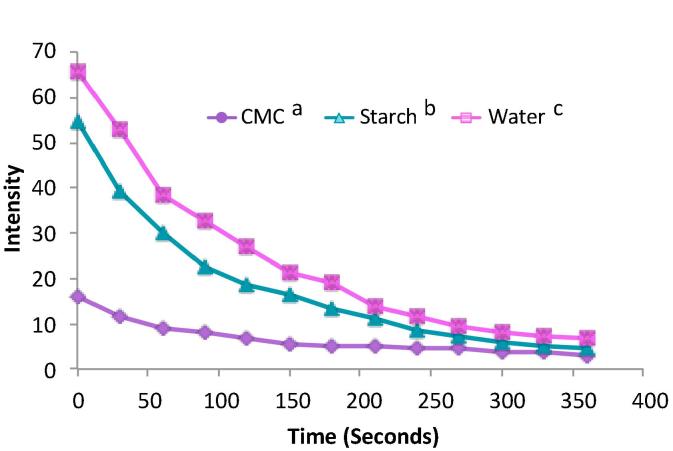
Progressive profiling data for *Saltiness*. Each data point represents the mean for the 11 panellists and their duplicate tests. Error bars are not included in this graph as there is large individual variation in scores over the time period. The letters next to the sample key represent statistically significant groupings. Different letters represents a significant difference of p ≤ 0.05.

Scores of saltiness intensity were recorded several times over 6 min using standard line scales (Fig. S4). The results for this attribute show that all three samples decreased in the intensity of saltiness over time (Fig. 4). Saltiness perception was significantly higher in the water samples compared to starch over time (p < 0.05), however, after 2 min the difference between them became non-significant (p > 0.05). The saltiness of the CMC sample was reduced compared to starch (p < 0.01) and water (p < 0.001) initially, and this difference persisted over time (Fig. 4). Saltiness intensity was significantly higher for water samples compared to samples with CMC at all time points. The starch samples were significantly higher than CMC until 480 s after which the scores were not significantly different.

There are various factors to consider with salt taste perception such as viscosity, matrix-tastant interactions and adaptation. An increase in viscosity is known to reduce the diffusion of tastant molecules as predicted by the Stokes-Einstein and Wilke-Chang equations (Wilke & Chang, 1955) and subsequently decrease taste perception in foods (Christensen, 1980, Cook et al., 2003, Hollowood et al., 2002, Kokini et al., 1982). Furthermore, interactions between ionic thickeners can slow the diffusion of charged molecules and recent research suggests that sodium ion availability from food matrices is the most important factor to consider for salt taste (Scherf, Pflaum, Koehler, & Hofmann, 2015). The interactions are often due to adsorption, entrapment in microregions, complexation, encapsulation, and hydrogen bonding (Kinsella, 1989, Scherf et al., 2015). Therefore, if the tastant is being chemically or physically prevented from diffusing out of the food matrix to reach taste bud receptors, then this will stunt perception.

How well the matrix mixes with saliva has also been proposed as an explanation to why starch does not stunt perception as much as random coil polysaccharides (Ferry et al., 2006). Another possibility is that adaptation effects are artificially turning down the saltiness signal, however, this would be more likely with stronger tasting solutions than weaker more prolonged taste.

The most likely explanation for the results found in this study, however, is the anion effect stunting the perception of sodium (Ye, Heck, & DeSimone, 1991). Although sodium ions themselves are responsible for activating taste cells, the anion associated with it serves an important purpose. In order to be perceived, sodium ions must diffuse from the food matrix into the saliva where they then diffuse into the papillae where the taste bud receptor cells are located. The anion associated with the sodium cation has great implications on the amount of saltiness perceived from a given concentration of sodium. The anion effect explains why smaller anions such as chloride facilitate a salty perception and larger anions do not (Delwiche et al., 1999, Lewandowski et al., 2016, Ye et al., 1991). Briefly, as the sodium ions diffuse paracellularly to permeate the basolateral cells of a taste bud pore, anions larger than chloride will stay behind. This leads to the development of a transepithelial potential and hyperpolarisation of the taste cell. In the experiments in this study, the sodium levels were matched regardless of the counter ion so it makes sense that with CMC being the anion in this circumstance, the sodium ions will not produce a salty perception. (Ferry et al., 2006).

Due to these reasons, it is not clear whether the presence of a mucoadhesive would prolong the taste perception of saltiness as the salt perception was already lower with CMC at the start of the profile due to the large anion effect. The amount of added NaCl to the CMC samples was 25% of that added to the other samples. The average intensity (0–100) recorded by participants at the first scoring point was 16 for CMC, 55 for starch and 66 for water. This means that the CMC scores were 29% of the score for starch and 24% of the score for the water samples. It could therefore be argued if the amount of NaCl added was the same for all the samples then the CMC samples may not have had such a drop in intensity.

### 3.3 Sensory perception: adhesion & mouthcoating

**Fig. 5:**
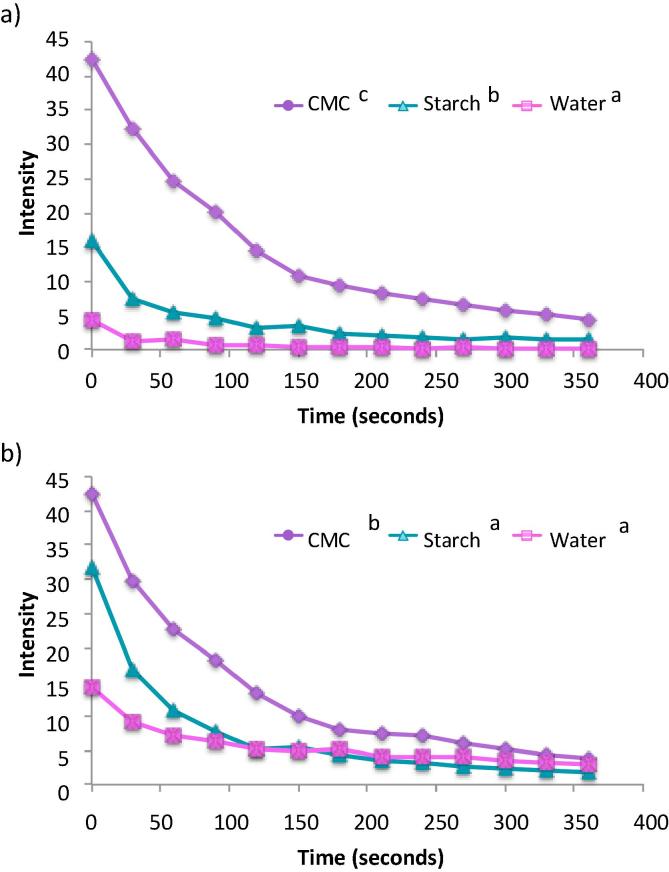
Progressive profiling data for a) *adhesion* and b) *mouthcoating*. Each data point represents the mean for the 11 panellists and their duplicate tests. Error bars are not included in this graph as there is large individual variation in scores over the time period. The letters next to the sample key represent statistically significant groupings. Different letters represents a significant difference of p < 0.05.

Panellists scored the attributes adhesion and mouthcoating at the same time as scoring the saltiness attribute. As these attributes are closely linked and have a similar response from the panellists, they will be discussed together. The scores for adhesion (Fig. 5a) and mouthcoating (Fig. 5b) were significantly higher for CMC samples overall compared to starch and water samples. During training the panel described the CMC samples as sticky and gummy whereas the starch was described as globular and gritty.

Immediately after swallowing and 30 s later the starch samples were perceived as more adhesive than water, which is unsurprising considering the added viscosity and bulk it imparts to a sample. CMC on the other hand scored significantly higher for adhesion up to 210 s for water (p > 0.05) and 480 s for starch (p > 0.05) (Fig. 5a). Adhesion scores were paralleled by mouthcoating scores (Fig. 5b), though starch scored higher for this attribute, presumably because it spreads throughout the oral cavity well but is extremely shear thinning (Fig. S3) so not particularly sticky when manipulated with the tongue. Mouthcoating scores for starch were initially higher than the water samples but dropped quickly, whereas CMC was significantly higher than the other samples for over 2 min after swallowing (Fig. 5b).

This is evidence that although panellists perceived that starch coated their mouth somewhat after swallowing, it was not adhesive in the same way as CMC. These results are in line with the *in vitro* retention experiments (Fig. 2), where CMC retained for longer on the tongue than starch. This prolonged adherence of the liquid formulation could be beneficial when delivering flavour molecules in liquid and semi solid food products.

### 3.4 *In vivo* salt retention

**Fig. 6:**
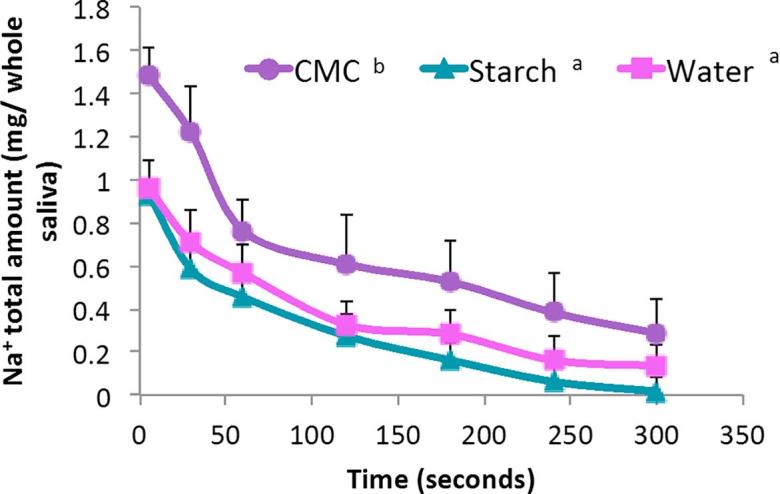
Amount of sodium present in participants’ whole saliva over 5 min. Each data point represents the mean of 15 (5 participants, 3 repeats) saliva collections analysis. Each sample presented to the participants contained 9 mg Na^+^, therefore, the first data point on this graph represents the residual sample left in the mouth after participants swallowed the sample. Error bars represent ±standard error mean. The letters next to the sample key represent statistically significant groupings. Different letters represents a significant difference of p < 0.05 using Bonferroni correction.

Five volunteers were used for retention experiments and each time point was carried out in triplicate for each sample. At set time points after consuming the sample, their whole saliva was extracted for analysis. This was to measure how much sodium was retained after the bulk of the sample had been swallowed. It was hypothesised that the presence of the mucoadhesive polysaccharide, CMC, would enhance the retention of sodium ions in the oral cavity. Fig. 6 shows the total amount of sodium present in the participants’ whole saliva at each time point. The total sodium amounts in the panellists’ saliva after tasting samples containing CMC were higher than the starch (p < 0.05) and water (p < 0.05) samples (Fig. 6). This suggests that the CMC samples were better at retaining the sodium ions because this polymer is more adhesive and, therefore, keeps the ions associated with it in the mouth for a prolonged period of time. This is supported by the results from the *in vitro* retention experiments (Fig. 2) and the sensory perception scores for adhesion and mouthcoating (Fig. 5a & b). Although the perception of sodium was stunted due to the anion effect, the actual amounts of the tastant were higher and retained for longer.

CMC is an ionic polysaccharide and this ionic nature lends itself to mucoadhesion due to ionic and hydrogen bond formation and Van der Waals interactions with the oral mucosa. However, the drawback of this from a nutritional perspective is that CMC inherently has sodium associated with the negatively charged carboxylic groups. This is the case for many ionic polysaccharides and therefore adding these types of polysaccharides to foods will increase the sodium content without necessarily adding to the salty taste. In this study, the sodium content of the samples were matched in order to ascertain whether the inherent sodium in CMC would elicit a salt response and prolong this perception over time. However, the amount of sodium already in the CMC samples meant that the amount of NaCl added to the CMC samples was a quarter of that which was added to the other samples. If there were equal amounts of NaCl added then the anion effect would be minimized and perhaps there would be a prolonged perception of saltiness. Of course, this would then mean that there was much more sodium in those samples making it less ideal from an application point of view.

As mucoadhesion is correlated with viscosity (Fig. 1), a non-ionic polysaccharide could be used to overcome the excess sodium issue. The mucoadhesive strength of polymers does not solely rely on viscosity; however, in liquid and semi-solid formulations this may be an overriding factor. The rheological behaviour is also an important consideration as CMC is relatively less shear thinning compared to starch, which may explain the retention further. The force required to remove the CMC samples may need to be higher than for the starch for example. Therefore, similar cellulose derivatives that are non-ionic such as hydroxypropyl methylcellulose may be retentive due to the rheological behaviour but will not have the associated sodium with them. Liquid mucoadhesion is heavily influenced by viscosity, the more viscous a sample is the more resistant it is to force. It is, therefore, difficult to control for viscosity in such experiments as most polysaccharides that can form viscous solutions are also going to exert some mucoadhesive strength. Furthermore, polymer chain flexibility that facilitates chain entanglement is inherently related to mucoadhesion, so this further complicates the endeavour to find a polymer that exhibits this characteristic in solution and is not mucoadhesive. Therefore, starch was chosen as one of the few polymeric substances that thicken solutions without forming an interconnecting polymer chain network.

Although water was not statistically different to starch at retaining sodium ions (p > 0.05) there was a general trend that more sodium was retained in the water samples over the different time points (Fig. 6). This could be explained due to the viscosity of starch; some of the sodium ions would reside in the starch matrix and be swallowed in the bolus as it is not mucoadhesive, thus reducing the amount left in the mouth. As there is no bolus formation in the water samples and water poses no physical barrier to the mucosa, the sodium ions are free to diffuse into the taste bud pores to be perceived and remain in the mouth.

Individual differences in salivary flow, composition and viscosity would likely have an impact on the retention of the sample. For example, a high salivary flow would dilute the sample and reduce the relative amount of polysaccharide chains interacting with the mucosa, therefore, reducing the mucoadhesive strength. This data was not collected during these experiments but would an interesting follow up study to link individual saliva properties, mucoadhesion and the impact on sensory perception of food containing mucoadhesives.

## 4 Conclusions

The results from this study show that a formulation containing mucoadhesive CMC prolongs the adherence of the matrix to the mucosa; *in vitro* and *in vivo* studies show that it also retains the model tastant, sodium, within it for longer than starch and water matrices. However, this study found that, due to the large anion effect, the perception of the retained sodium was diminished. This study suggests that mucoadhesive matrices may result in a controlled release of flavour compounds after consumption when the anion effect is not an issue. Although there is much work needed in this area to better understand the role of mucoadhesion in foods, this evidence can be used to design foods to sustain delivery of flavour ingredients.
