# Supplementary material for: Mucoadhesive polysaccharides modulate sodium retention, release and taste perception
Source: Food Chem. 2018 Feb 1;240:482–9. doi: 10.1016/j.foodchem.2017.07.134 (PMC5625848; doi:10.1016/j.foodchem.2017.07.134)
Supplement: Supplementary data — Supplementary Figures S1–S4. [file mmc1.docx]

Supplementary information

Figure S1 shows the calibration curve for flame photometry. The same standards were used for each experiment set to keep consistency. The flame photometer was set to 100 at the highest point of the calibration and then each standard was measured for intensity.

Figure S1. Calibration curve for flame photometry

Figure S2 a) shows the frequency sweeps of CMC at increasing concentrations. The polysaccharide solutions are non-shear thinning until the concentration of 5.5% is reach where shear-thinning behaviour is demonstrated. S1 b) shows the exponential increase in viscosity as the concentration is increased. At low concentrations (1.4 – 4%) the relationship is relatively linear but increases exponentially there after.

a)

b)

Figure S2. a) frequency sweep of different concentrations of CMC. b) Complex viscosity (η*) of the different concentrations at 50 rad/s.

S3 shows the frequency sweeps for the samples used in perception and retention experiments. Starch is very shear thinning where it appears to have a high viscosity under limited shear but the viscosity quickly decreases upon higher shear. At 50 rad/s there is no statistical significant difference between the viscosities of the two samples. There is clearly a different behaviour of the two samples and is mostly due to the internal structures that are formed with CMC forming interconnecting chain networks and starch swelling in granules.

Figure S3. Complex viscosity (η*) frequency sweeps for CMC and Starch samples.

Figure S4 is an example of the line scales used during the sequential profiling experiment. Each assessor used a computer set up with Compusense software. A test was designed so that the assessor would rate the attributes dependent on the intensity they perceived. After assessing these attributes, there was a 20 second break between until assessors were prompted to score the same attributes. Panellists took and average of 10 seconds to score the 3 attributes and therefore the average tie between scoring was 30 seconds.

Figure S4. Exemplary line scales used during sequential profiling of samples. Panellists would score along the line appropriately as they felt the intensity of each attribute for the particular sample. This process was repeated every 30 seconds for 6 minutes.
